# Unique Transcriptome Signature Distinguishes Patients With Heart Failure With Myopathy

**DOI:** 10.1161/JAHA.120.017091

**Published:** 2020-09-05

**Authors:** Talia Caspi, Sam Straw, Chew Cheng, Jack O Garnham, Jason L. Scragg, Jessica Smith, Aaron O. Koshy, Eylem Levelt, Piruthivi Sukumar, John Gierula, David J. Beech, Mark T. Kearney, Richard M. Cubbon, Stephen B. Wheatcroft, Klaus K. Witte, Lee D. Roberts, T. Scott Bowen

**Affiliations:** ^1^ Leeds Institute of Cardiovascular and Metabolic Medicine University of Leeds United Kingdom; ^2^ School of Biomedical Sciences Faculty of Biological Sciences University of Leeds United Kingdom

**Keywords:** chronic heart failure, metabolism, mitochondria, skeletal muscle, Metabolism, Heart Failure

## Abstract

**Background:**

People with chronic heart failure (CHF) experience severe skeletal muscle dysfunction, characterized by mitochondrial abnormalities, which exacerbates the primary symptom of exercise intolerance. However, the molecular triggers and characteristics underlying mitochondrial abnormalities caused by CHF remain poorly understood.

**Methods and Results:**

We recruited 28 patients with CHF caused by reduced ejection fraction and 9 controls. We simultaneously biopsied skeletal muscle from the pectoralis major in the upper limb and from the vastus lateralis in the lower limb. We phenotyped mitochondrial function in permeabilized myofibers from both sites and followed this by complete RNA sequencing to identify novel molecular abnormalities in CHF skeletal muscle. Patients with CHF presented with upper and lower limb skeletal muscle impairments to mitochondrial function that were of a similar deficit and indicative of a myopathy. Mitochondrial abnormalities were strongly correlated to symptoms. Further RNA sequencing revealed a unique transcriptome signature in CHF skeletal muscle characterized by a novel triad of differentially expressed genes related to deficits in energy metabolism including adenosine monophosphate deaminase 3, pyridine nucleotide‐disulphide oxidoreductase domain 2, and lactate dehydrogenase C.

**Conclusions:**

Our data suggest an upper and lower limb metabolic myopathy that is characterized by a unique transcriptome signature in skeletal muscle of humans with CHF.

People with chronic heart failure (CHF) caused by reduced left ventricular ejection fraction (ie, heart failure with reduced ejection fraction) develop significant skeletal muscle abnormalities underpinned by impaired mitochondrial energy metabolism, which more closely correlate to symptoms than to the degree of cardiac dysfunction.^1^ Improved skeletal muscle mitochondrial function in CHF is associated with alleviation of symptoms and improved quality of life, and is considered a worthy therapeutic target.^2^ However, controversy remains as to whether muscle mitochondrial abnormalities in CHF are central to the disease process or occur secondary to detraining.^3^ Most studies in patients with CHF have used thigh biopsies to investigate mitochondrial dysfunction,^1^ while few studies have used alternative sample sites that are unlikely impacted by detraining. Yet, various upper limb muscles (eg, pectoralis major and diaphragm) in patients with CHF exhibit histological perturbations similar to (or even greater than) the lower limbs (eg, vastus lateralis).^1^ The pectoralis major also demonstrates mitochondrial dysfunction^4^ alongside muscle atrophy that independently predicts mortality in patients with CHF.^2^ This evidence provides support for a myopathy in patients with CHF independent of detraining.

The mechanisms underpinning mitochondrial dysfunction in CHF remain poorly defined. Putative mechanisms specific to the disease include impaired energy homeostasis via dysregulation in high energy phosphates (eg, ATP, ADP, and AMP), activation of atrophic signaling networks involved in protein degradation, elevated inflammatory cytokines and reactive O_2_ species that disrupt cellular homeostasis, and abnormal microvascular homeostasis.^1^ Yet, many of these hypotheses have been investigated in animal models rather than human tissue. Whole transcriptome RNA sequencing (RNA‐seq) has been applied in people with diabetes mellitus,^5^ critical illness,^6^ peripheral arterial disease,^7^ disuse,^8^ and aging^9^ to reveal novel molecular mechanisms of skeletal muscle abnormalities. However, to our knowledge, the molecular signature of skeletal muscle in patients with CHF has yet to be investigated.

Here, we recruited a total of 28 patients with CHF and 9 controls. We simultaneously biopsied skeletal muscle from the pectoralis major (PM) in the upper limb and from the vastus lateralis (VL) in the lower limb. Mitochondrial function was phenotyped in myofibers from both sites using high‐resolution respirometry to determine whether upper and lower limb impairments were present and their association with symptoms. We then applied RNA‐seq to identify underlying molecular abnormalities of muscle dysfunction in patients with CHF. Overall, we found that patients with CHF present with a similar degree of mitochondrial dysfunction in both the PM and VL, indicative of a myopathy. RNA‐seq analysis identified a triad of differentially expressed genes related to impaired mitochondrial energy metabolism. We believe our data reveal, for the first time, a unique transcriptome signature in skeletal muscle of humans with CHF.

## Methods

The data that support the findings of this study are available from the corresponding author upon reasonable request. Clinical characteristics and medications for all patients are presented in Tables S1 and S2, respectively. Patients were allocated to control (n=9) or CHF (n=28) groups. Controls had no clinical evidence of CHF: left ventricular ejection fraction ≥50% and no previous diagnosis of left ventricular systolic dysfunction. Patients with CHF had stable symptoms of CHF (>3 months on medical therapy) and a left ventricular ejection fraction <50% as confirmed by transthoracic echocardiography (following current European Society of Cardiology guidelines). All participants were indicated for device therapy with either a permanent pacemaker, implantable cardioverter‐defibrillator, or cardiac resynchronization therapy device according to current indications. Patients with CHF performed a peak symptom‐limited exercise test to volitional exhaustion on a cycle ergometer for determination of peak pulmonary O_2_ uptake (${\dot{V}O}_{2\text{peak}}$). Exclusion criteria included inability to provide informed consent because of cognitive dysfunction or the presence of previous diagnoses with other potentially confounding comorbidities, such as chronic obstructive pulmonary disease or cancer. All patients provided written informed consent and all procedures were conducted in accordance with the Declaration of Helsinki after receiving local institute ethical approval (11/YH/0291).

### Muscle Biopsy, Mitochondrial Respiration, and RNA‐Seq

Muscle samples were taken from 2 sites in participants: the upper limb (PM) and lower limb (VL). The PM sample was obtained during routine device implantation procedures, while on the same day the VL sample was from the right thigh. One piece of fresh muscle sample was used for the assessment of mitochondrial respiration,^4^ while another small portion was rapidly frozen in a subset of patients (controls=3, CHF=6) for subsequent RNA‐seq analysis.^10^ See Data S1 for expanded details.

### Statistical Analysis

Assumption of homogeneity of variance was confirmed using Levene test, while Shapiro‐Wilk and Kolmogorov–Smirnov normality tests confirmed normal (Gaussian) distribution. Unpaired Student *t* test was used to compare between‐group differences, while paired Student *t* test was used for within‐group comparisons for continuous data and chi‐square test for categorical data. Further, ANCOVA was performed to adjust for the variables of age and sex. Pearson correlation coefficient (*r*) was performed to assess the association between mitochondrial respiration at different muscle sites and ${\dot{V}O}_{2\text{peak}}$. Continuous data are expressed as mean±SEM and categorical data as number (percentage). Statistical significance was accepted as *P*<0.05.

## Results

### Mitochondrial Myopathy in Patients With CHF

**Figure 1:**
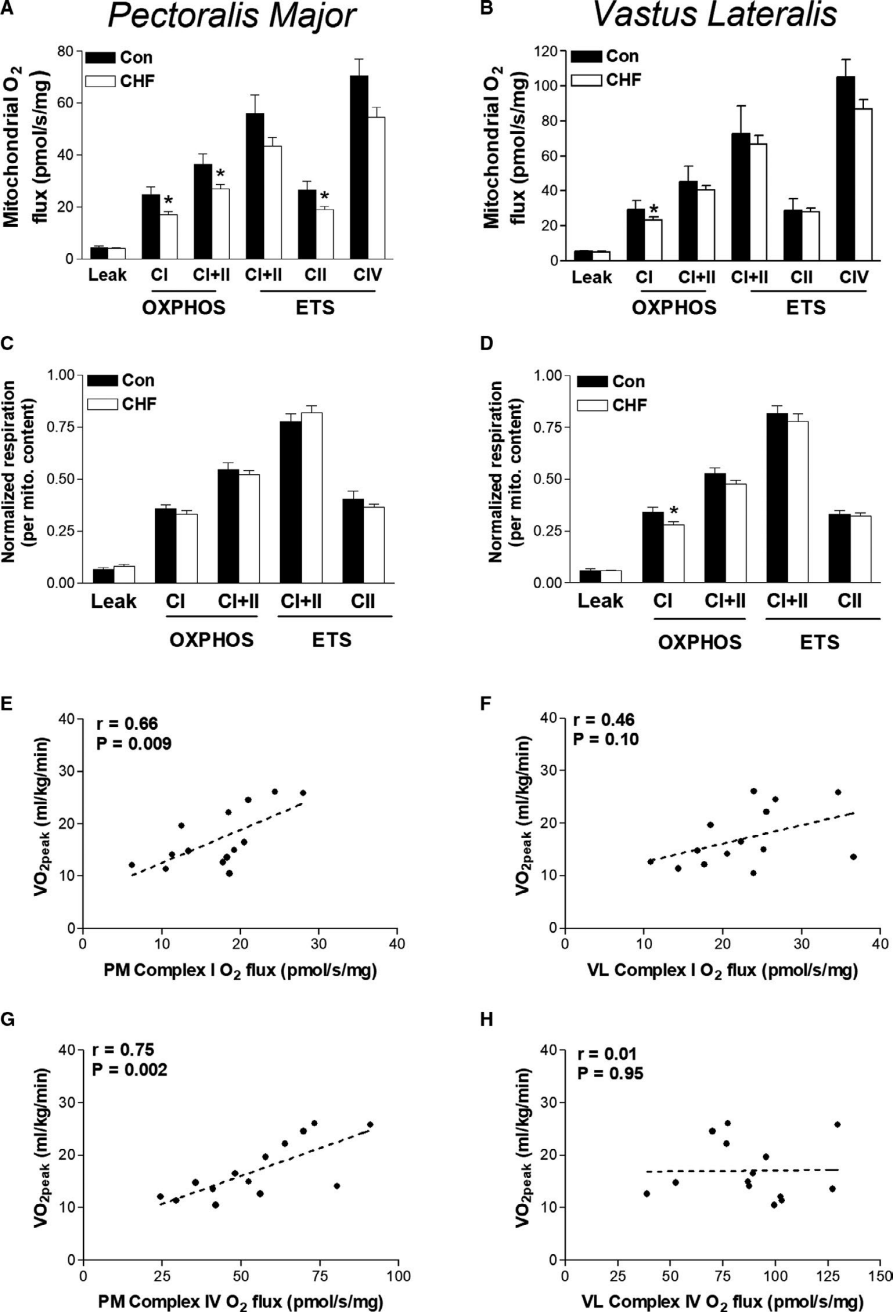
Skeletal muscle mitochondrial respiration is impaired in patients with heart failure and correlated to exercise intolerance. Mitochondrial O_2_ flux measured in myofibers biopsied from pectoralis major (PM) and vastus lateralis (VL) of patients with chronic heart failure (CHF) (n=22) and controls (n=6). Mitochondrial respiration in the PM and VL is presented between groups in both absolute units (**A** and **B**) and normalized to mitochondrial content (**C** and **D**). Correlations between exercise intolerance (ie, VO_2peak_) and complex I respiration (**E** and **F**) and mitochondrial content (ie, complex IV) in CHF (**G** and **H**). Leak (L_I_), complex I (P_I_), complex I and II (P_I+II_ and E_I+II_), complex II (E_II_) and complex IV (C_IV_) measured under oxidative phosphorylation (OXPHOS; P), and electron transport system (ETS; E) supported respiration. *P<0.05 between‐group comparisons. Correlation coefficient (*r*). Data are mean±SEM.

We first aimed to confirm the presence of skeletal muscle mitochondrial dysfunction in patients with CHF in both upper and lower limb muscle sites. Based on published data, we hypothesized that mitochondrial dysfunction would occur to a greater degree in the lower compared with the upper limbs. We analyzed mitochondrial O_2_ consumption in permeabilized fibers of PM and VL from patients with CHF using high‐resolution respirometry. Mitochondrial O_2_ flux was, on average, lower by 15% to 20% in patients with CHF among respiratory states (Figure 1A and 1B). ADP‐stimulated mitochondrial complex I respiration was lower in both PM (*P*=0.028) and VL (*P*=0.015) by 25% and 28%, respectively (Figure 1A and 1B). Convergent complex I and II respiration (*P*=0.047) and complex II respiration (*P*=0.037) were also significantly lower in the PM of patients with CHF compared with controls by ≈20% (Figure 1A). However, after adjusting for confounding variables (specifically age and sex) between the 2 groups, differences in mitochondrial respiration were not detected (*P*>0.05). Mitochondrial respiration is determined by both organelle quantity and quality. A lower mitochondrial content is most often reported in patients with CHF.^3^, ^11^ We therefore assessed intrinsic mitochondrial respiration after normalization to complex IV activity (a proxy for mitochondrial mass).^4^ Here, we observed fewer differences between patients and controls (Figure 1C and 1D), with only VL showing an 18% lower complex I respiration versus controls (*P*=0.048, Figure 1D)

### Whole‐Body Mitochondrial Impairments Correlate to Symptoms

Having identified that both upper and lower muscles of patients with CHF experience similar reductions in mitochondrial respiration, we further characterized their clinical importance. We correlated mitochondrial deficits in PM and VL to the gold‐standard measure of whole‐body exercise intolerance: peak pulmonary O_2_ uptake (VO_2peak_). We focused on complex I and complex IV respiration, given our earlier findings. Complex I respiration was well correlated with VO_2peak_ in PM but less so in VL (Figure 1E and 1F), while complex IV respiration correlated well with measures in PM but not VL (Figure 1G and 1H).

### Lower Limbs Demonstrate Greater Oxidative Capacity

**Figure 2:**
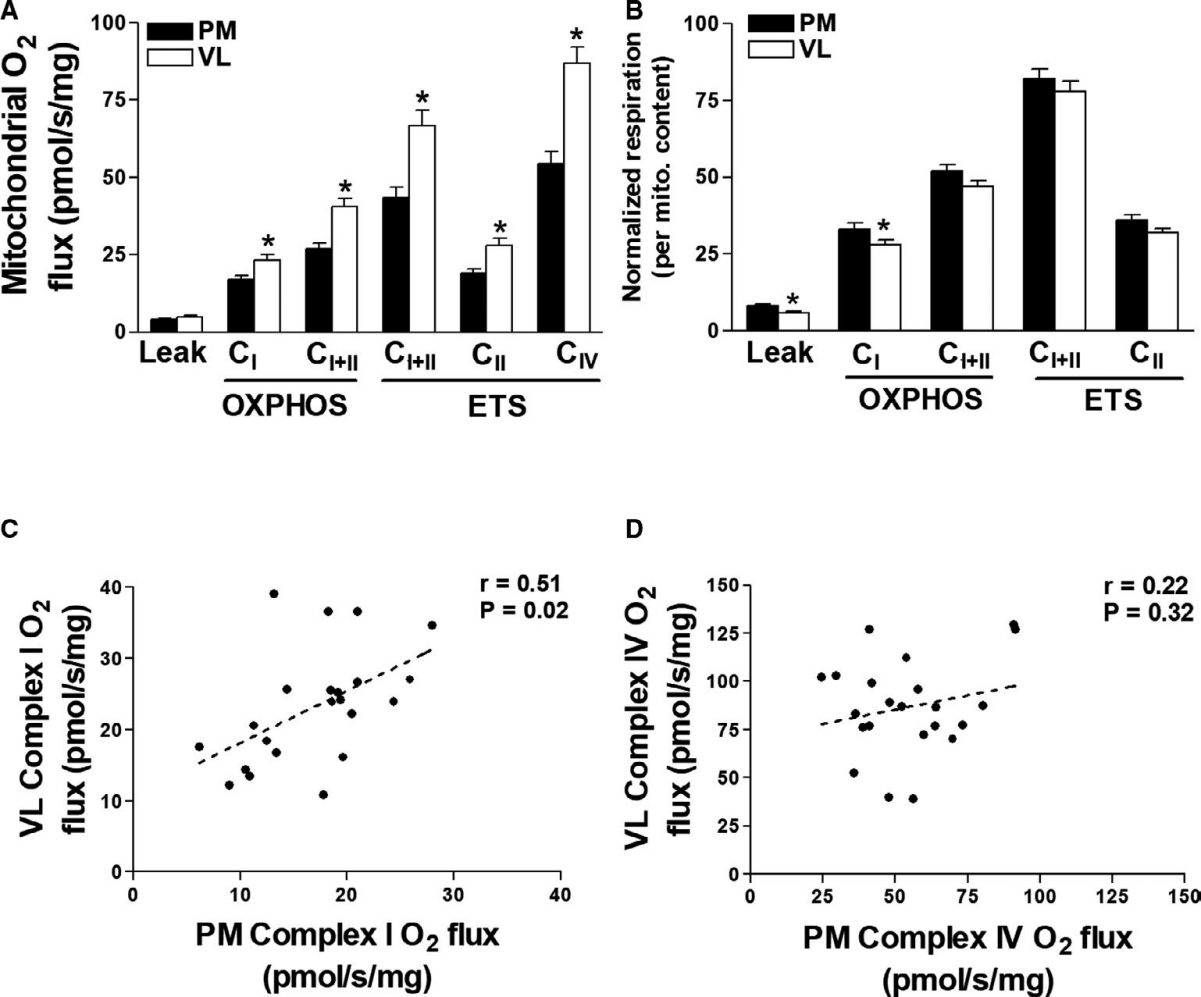
Mitochondrial respiration varies between upper and lower limb skeletal muscle in patients with heart failure. Pectoralis major (PM) and vastus lateralis (VL) mitochondrial respiration measured in myofibers of patients with chronic heart failure (CHF) (**A**) and corrected for mitochondrial content (**B**). Correlations between VL and PM complex I function (**C**) and complex IV function (mitochondrial content) (**D**). Leak (L_I_), complex I (P_I_), complex I and II (P_I+II_ and E_I+II_), complex II (E_II_) and complex IV (C_IV_) measured under oxidative phosphorylation (OXPHOS; P) and electron transport system (ETS; E) supported respiration. *P<0.05. Correlation coefficient (*r*). Data are mean±SEM.

The PM and VL demonstrated distinct phenotypes in correlations between mitochondrial mass and exercise intolerance. Therefore, we compared mitochondrial respiration between PM and VL muscle sites in patients with CHF for mechanistic insight. Compared with PM, patients with CHF demonstrated greater mitochondrial O_2_ flux in VL across respiratory states. Both complex I and complex II respiration were ≈30% higher in VL versus PM (*P*<0.001). Mitochondrial content, as assessed by complex IV respiration, was also higher in VL versus PM by ≈40% (Figure 2A). We therefore normalized PM and VL respiration to mitochondrial content to assess intrinsic respiration. PM had higher intrinsic mitochondrial respiration at leak (*P*=0.01) and complex I (*P*=0.02) compared with VL (Figure 2B); however, no differences between the respiratory control ratio (RCR) was detected (5.0±0.6 versus 5.4±0.4, *P*>0.05). Furthermore, complex I respiration was correlated between upper and lower limb muscles (*r*=0.51, *P*=0.02; Figure 2C). This was not observed for complex IV respiration (*r*=0.22, *P*=0.32; Figure 2D).

### RNA‐Seq Analysis in Human Heart Failure Skeletal Muscle

**Figure 3:**
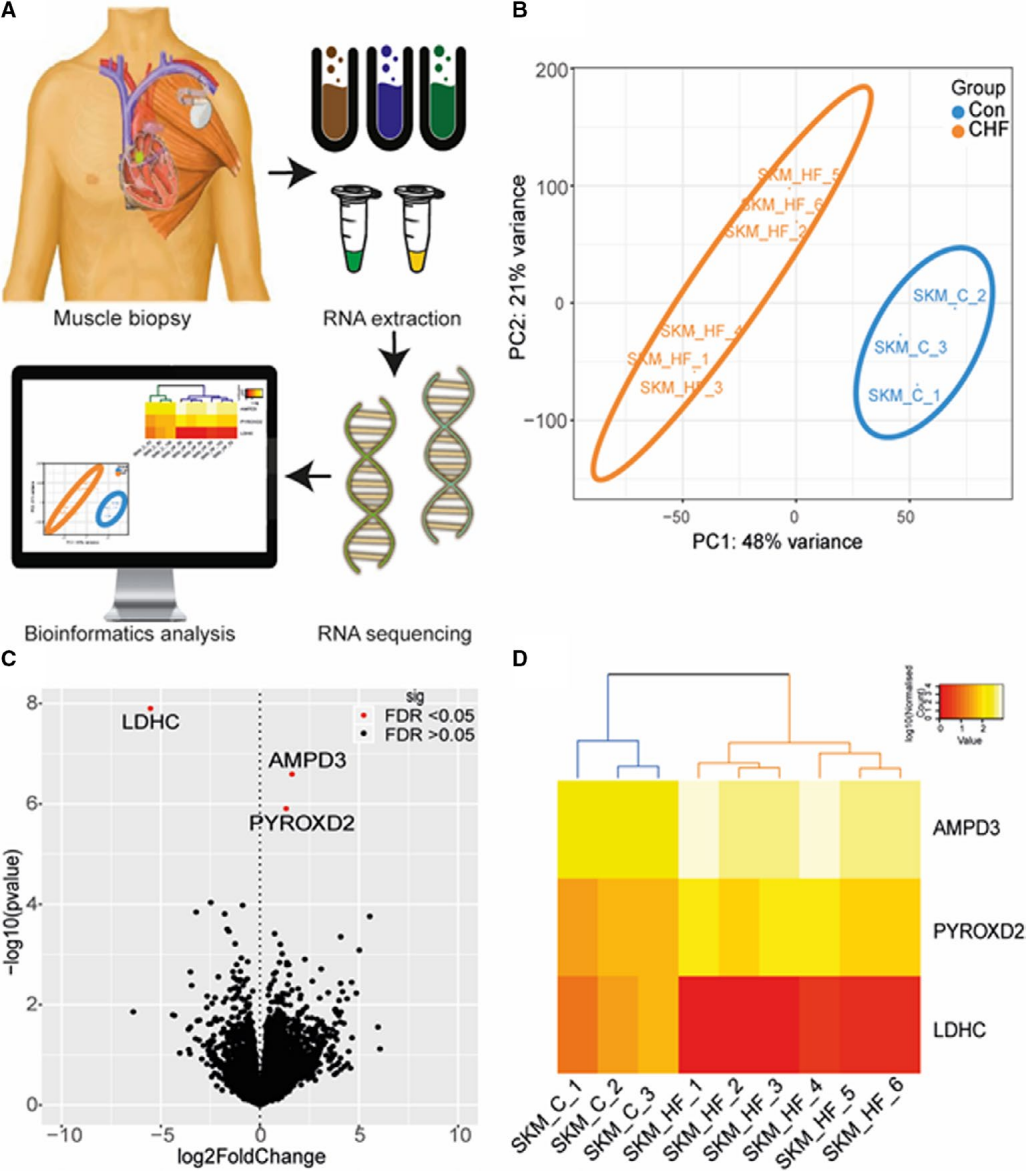
Patients with heart failure are characterized by a distinct skeletal muscle transcriptome signature. RNA‐sequence analysis workflow (**A**), where 9 pectoralis major (PM) human biopsies were obtained (n=3 controls; n=6 chronic heart failure [CHF]) and RNA samples proceeded with library preparation and RNA sequencing. Bioinformatics processing was performed on the raw reads and identified the significantly regulated genes in CHF. Partial least squares discriminant analysis (PLS‐DA) plot of expression profiles for 18 426 genes among the 9 patients, CHF (orange) controls (blue) (**B**). Volcano plot showing the 3 significantly regulated genes (red dots), including AMP deaminase 3 (AMPD3) and pyridine nucleotide‐disulphide oxidoreductase domain 2 (PYROXD2) upregulated in CHF and lactate dehydrogenase C (LDHC) downregulated (**C**). Heat map of differentially expressed genes (DEGs) in CHF and control samples (**D**). DEGs in the heat map were clustered based on a hierarchical algorithm implemented in *“*pheatmap*.”* Heat map was generated with log_10_‐normalized reads derived from *DESeq2*. Color scale represents log_10_‐transformed normalized reads.

We have demonstrated that mitochondrial dysfunction induced by CHF occurs in both the upper and lower limbs that closely correlates to symptom severity. Next, we used high‐throughput RNA‐seq to interrogate the molecular alterations accompanying skeletal muscle impairments in CHF (Figure 3A). To our knowledge, our study is the first to perform whole‐transcriptome sequencing in skeletal muscle in humans with CHF. RNA‐seq was performed on PM biopsy from 9 participants (3 controls and 6 CHF, Table S3), which generated ≈16 million 75 bp single‐end reads for each individual (Table S4). Only uniquely mapped reads were retained for downstream differential expression analysis. Overall, ≈92% of the reads mapped uniquely to the human reference genome (GRCh38). RNA integrity number scores were consistently high for all samples (RNA integrity number >7.5). The read counts were normalized using median of ratios as implemented in *DESeq2*. The normalized gene expression was expressed as read counts for all 18 426 genes (Table S5). To determine whether expression profiles among the 9 patients could discriminate them based on the disease status, the data set was analyzed using partial least squares discriminant analysis. Partial least squares discriminant analysis plot distinguished 2 groups of individuals: controls and patients with CHF (Figure 3B). The first component accounted for 48%, and the second component accounted for 21% of the variance in the partial least squares discriminant analysis model. Interestingly, our transcriptome analysis also appeared to identify 2 distinct clusters of patients with CHF (Figure 3B): cluster 1 (n=3; patients 1, 3, and 4) and cluster 2 (n=3; patients 2, 5, and 6). When we further analyzed demographic and clinical characteristics of these 2 clusters (Table S6) we found only left ventricular systolic function to be different between the 2 groups, with a lower value in cluster 2 versus cluster 1 (14%±1% versus 38%±5%, *P*<0.01; Table S6). Taken together, these data may indicate that the degree of left ventricular systolic dysfunction is a potential determinant of the skeletal muscle transcriptome in patients with CHF.

### Distinct Transcriptome Signature in Skeletal Muscle of CHF

From a total of 18 426 genes identified, 3 genes related to energy metabolism were differentially expressed genes between control and CHF at a fold change >1.5 and false discovery rate threshold <0.05 (Figure 3C). Two genes were upregulated in CHF: AMP deaminase 3 (AMPD3) (log_2_‐fold change=1.62, false discovery rate=0.0023) and pyridine nucleotide‐disulphide oxidoreductase domain 2 (PYROXD2) (log_2_‐fold change=1.33, false discovery rate=0.0077), while lactate dehydrogenase C (log_2_‐fold change=−5.52, false discovery rate=0.0002) was downregulated (Figure 3C). Hierarchical clustering illustrated on the heat map using the 3 differentially expressed genes clearly show 2 distinct branches. In patients with CHF we found that lactate dehydrogenase C was consistently downregulated among the 6 patients, while AMPD3 and PYROXD2 were upregulated (Figure 3D). Overall, whole‐transcriptome sequencing revealed a novel triad of metabolic genes that are dysregulated in skeletal muscle from patients with CHF.

## Discussion

This is the first study to demonstrate that skeletal muscle from patients with CHF is characterized by a unique transcriptome signature that underpins an upper and lower limb metabolic myopathy closely linked to worse symptoms. We identified a triad of genes dysregulated in CHF that are involved in ATP homeostasis. These findings have important implications for our understanding of the link between muscle abnormalities and symptoms in patients with CHF.

### Is There a CHF‐Associated Myopathy?

Our data highlight that both PM and VL in patients with CHF show a similar degree of loss in mitochondrial respiratory function (Figure 1A and 1B). This upper and lower limb dysfunction appears primarily driven by a decrease in mitochondrial volume and dysfunction of complex I respiration, which is likely a major contributor exacerbating symptoms of fatigue in many patients. We speculate that this could reflect a mitochondrial myopathy that is independent of disuse, which may exacerbate symptoms of exercise limitation (Figure 1E through 1H). Our findings further revealed that while mitochondrial capacity is higher per muscle mass in the leg, O_2_ flux normalized per mitochondrial mass (ie, intrinsic function) is higher in the chest muscle (Figure 2A and 2B). Thus, it seems that the VL has higher mitochondrial mass compared with upper limb muscles, which is likely a consequence of higher metabolic demands during daily activities. This may be important because, unlike the VL, the PM did not demonstrate reductions in intrinsic mitochondrial function versus healthy controls. The close association between O_2_ flux measurements taken from chest and leg muscle further provides validation for the PM being an alternative site for investigating muscle dysfunction in patients with CHF, which aligns with recent data showing that PM muscle volume independently predicts mortality.^2^

### Is Lactate Dehydrogenase C Limiting Anaerobic Metabolism?

Patients with CHF have an increased reliance on anaerobic metabolism for a given exercise work load compared with matched controls,^12^ which is caused by significant limitations to mitochondrial oxidative metabolism (Figure 1). Our RNA‐seq analysis showed that the glycolytic enzyme lactate dehydrogenase C was severely downregulated by 46‐fold in patients with CHF versus controls (Figure 3C and 3D), which is consistent with earlier work^3^, ^12^ and indicates disruptions to NAD^+^/NADH homeostasis in CHF skeletal muscle. Overall, these data implicate a specific impairment at the transcriptional level of the lactate dehydrogenase enzyme and identify components of both oxidative and glycolytic metabolic machinery to be perturbed in CHF.

### Is AMPD3 a Mediator of Impaired Metabolism and Fiber Atrophy?

The AMPD3 gene showed the highest upregulation in CHF by 3‐fold (Figure 3C and 3D), which indicates a shift toward a greater reliance on the AMP deaminase reaction in skeletal muscle of patients with CHF. AMPD3 catalyzes the first and rate‐limiting step of the purine nucleotide cycle, highlighting its role as a key enzyme muscle energetics and amino acid catabolism. Under metabolic stress, AMP levels increase as ADP is converted to ATP and AMP via the adenylate kinase reaction, which maintains a high ATP/ADP ratio. AMP is then irreversibly deaminated by AMP deaminase to inosine monophosphate and ammonia. In vivo evidence confirms intramuscular concentration of ADP are higher and Phosphocreatine (PCr) and pH are lower for given work rates in patients with CHF,^12^, ^13^ which could explain our transcriptome findings that AMPD3 expression is increased to compensate under metabolic stress where ATP is low (Figures 1, 2, 3).^12^, ^13^ AMP activates glycolytic metabolism and this mechanism is likely impaired given the capacity of glycolytic enzymes is lower in muscle from patients with CHF.^3^, ^12^ Increased reliance on AMP deaminase would increase inosine monophosphate and ammonia formation while lowering the total adenine nucleotide pool thereby exacerbating muscle fatigue. Elevated levels of inosine monophosphate are converted back to AMP by combining with the amino acid aspartate via the purine nucleotide cycle. Increased expression of AMPD3 in skeletal muscle of patients with CHF may reflect a fuel shift toward increased catabolism of amino acids and thus provide a novel mechanism linking metabolic dysregulation and muscle wasting in patients with CHF given AMPD3 is upregulated in various atrophic conditions in animal models such as disuse, starvation, cancer, and denervation.^14^ Here, we translate this to humans with CHF.

### Does PYROXD2 Link to Mitochondrial Dysfunction and Myopathy?

PYROXD2 was upregulated by 2.5‐fold in patients with CHF (Figure 3C and 3D). The physiological role of PYROXD2, particularly in skeletal muscle, remains largely unclear but recent data show that genetic variants in its family member PYROXD1 induces early‐onset myopathy and limb girdle muscular dystrophy characterized by abnormal myofibrils, nuclei, and mitochondria.^15^ PYROXD2 is part of a pyridine nucleotide‐disulfide oxidoreductase domain‐containing protein family that is involved in redox and energy balance. Pyridine nucleotidedisulphide reductases are flavoproteins (FAD binding) that catalyze the pyridine nucleotide (NAD/NADH)‐dependent reduction of cysteine residues in their substrates,^15^ implicating a role for impaired NAD^+^/NADH homeostasis. PYROXD2 (also known as YueF) can also induce the expression of the transcription factor p53,^16^ with the latter known to promote fiber atrophy in various conditions including limb immobilization and cancer cachexia.^17^ Overall, these studies identify PYROXD2 as a potential regulator of muscle homeostasis in patients with CHF by controlling metabolic homeostasis, mitochondrial function, and fiber atrophy via a redox‐dependent pathway.

### Study Limitations

Our analyses were performed in a relatively small sample size, mostly men and patients with a reduced ejection fraction, so should be viewed with caution when generalizing to the larger population of patients with CHF. In addition, our in situ mitochondrial respiration measurements may not reflect the in vivo muscle environment in patients, given some of the experimental settings such as O_2_ and substrate (eg, ADP) concentrations where unphysiological (as detailed elsewhere^18^), which may limit translation. We have also inferred that mitochondrial impairments in skeletal muscle are likely contributing to symptoms of exercise intolerance to a greater degree than those related to cardiac dysfunction, as based on our correlative data. This suggestion is supported by clear data showing that exercise intolerance (ie, VO_2peak_) in patients with CHF poorly correlates with measures of cardiac dysfunction, while in contrast skeletal muscle impairments are closely associated.^19^ We also acknowledge that some variance in our results may be explained, at least in part, by other confounding factors such as sex, age, and levels of physical activity.^4^, ^20^ As such, future studies should carefully control for potential confounding factors that may have impacted our current results.

## Conclusions

We believe this is the first study to demonstrate that patients with CHF are characterized by a unique transcriptome signature in skeletal muscle that is underpinned by a metabolic myopathy in both the upper and lower limbs, which closely associated with worse symptoms. These findings have important implications for our understanding of the skeletal muscle pathology and its relation to symptoms in patients with CHF.

## Sources of Funding

This work was supported by Leeds Cardiovascular Endowment Fund. Straw by British Heart Foundation Clinical Fellowship. Roberts by Diabetes UK RD Lawrence Fellowship (16/0005382). Cubbon by British Heart Foundation Intermediate Research Fellow. Kearney British Heart Foundation Chair of Cardiology. Witte by National Institute for Health Research (NIHR) Clinician Scientist Award. Bowen by Medical Research Council UK (MR/S025472/1) and Heart Research UK (TRP 16/19).

## Disclosures

None.

## Supporting information


**Data S1**
**Tables S1–S6**
