# Supplementary material for: Unique Transcriptome Signature Distinguishes Patients With Heart Failure With Myopathy
Source: J Am Heart Assoc. 2020 Sep 5;9(18):e017091. doi: 10.1161/JAHA.120.017091 (PMC7727001; doi:10.1161/JAHA.120.017091)
Supplement: Supplementary file 1 — Data S1 Tables S1–S6 [file JAH3-9-e017091-s001.pdf]

Supplemental Material

Data S1.

Muscle biopsy

Samples were taken from two sites in participants, including from the upper limb (pectoralis major) and lower limb (vastus lateralis). *Pectoralis major* sample was obtained during routine device implantation procedures, while on the same day a *vastus lateralis sample* of the right thigh were obtained using the Bergström needle method modified to allow for suction. The sample region was cleaned beforehand with iodine and anaesthetised (1% lidocaine), while the wound was closed with surgical tape strips and a soft adhesive sterile dressing. Biopsies were taken within 3 months following study recruitment and collection of baseline clinical data. There were no complications or adverse events with any of the research procedures. One piece of muscle sample was immediately placed in 1 mL of ice-cold specialized preservation solution termed BIOPS[4] for assessment of mitochondrial respiration while another small portion was rapidly frozen in a small subset for RNA sequencing.

Mitochondrial function

Mitochondrial respiration was assessed *in situ* from saponin-permeabilized skeletal muscle fibres using high-resolution respirometry (Oxygraph-2K; Oroboros Instruments, Innsbruck, Austria), as previously described[4]. Briefly, in the following order: 1) complex I Leak respiration was determined by addition of glutamate (10 mM), malate (0.5 mM) and pyruvate (5 mM) (i.e., a measure of proton leak under non-phosphorylating conditions); 2) adenosine diphosphate (ADP; 2.5 mM) added to provide a measure of complex I oxidative phosphorylation (OXPHOS); 3) outer mitochondrial membrane integrity determined by addition of 10 μM cytochrome *c*; 4) succinate at 10 mM as a complex II substrate provided complex I+II OXPHOS; 4) 5 μM carbonyl cyanide 4-(trifluoromethoxy)-phenylhydrazone (FCCP) for maximal uncoupled complex I+II respiration; 5) complex I inhibitor rotenone at 0.25 μM provided uncoupled complex II respiration; and 6) 2.5 μM antimycin A as a complex III inhibitor for residual oxygen consumption (ROX) to calculate non-mitochondrial (background)

respiration, which was then used to normalise the data. Mitochondrial content was determined within the respirometer using a complex IV activity assay (a close proxy)[4] by the addition of 0.5 mM N,N,N',N'-Tetramethyl-p-phenylenediamine dihydrochloride (TMPD) as an artificial electron donor to complex IV in combination with 2 mM ascorbate to maintain TMPD in a reduced state. Absolute mitochondrial respiration was normalised to complex IV activity to provide an index of mitochondrial intrinsic function.

RNA isolation and sequencing

In a subset of patients (CON = 3; CHF = 6) biopsies were immediately transferred to a clean 2 ml screw-capped tube and flash-frozen in liquid nitrogen. A small piece of tissue (~30 to 50 mg) was added to a 2 ml microcentrifuge tube containing a stainless-steel bead and 1 ml TRIzol (Invitrogen, UK). The tissue was homogenized using a TissueLyser (Qiagen, UK) at frequency 25 Hz for 2 minutes. RNA was extracted from the homogenate with TRIzol using standard protocols. Nine RNA samples were treated with DNase I using the RNA Clean and Concentrator kit (Zymo Research, UK). The quality of the RNA was evaluated on a DeNovix DS-11 FX + Spectrophotometer / Fluorometer (DeNovix, USA) as per manufacturer's instruction. RNA was considered of high quality when A_{260}/A_{280} and A_{260}/A_{230} ratios achieved 2.0. Subsequently, Agilent 2100 Bioanalyzer (Agilent Technology, USA) was used to measure RNA integrity number (RIN). Nine RNA samples achieved RIN value >7.5 and proceeded with RNA sequencing (RNA-seq). RNA-seq was performed using the TruSeq RNA library preparation kit at the Cambridge Genomics Services. The RNA-seq libraries were sequenced on the NextSeq 500 platform in a single lane, generating ~16 million single-end 75 bp reads for each sample (Table 4). All the protocols were carried out according to the manufacturer's recommendations. The raw reads were deposited at the ArrayExpress domain (https://www.ebi.ac.uk/arrayexpress/) under the accession E-MTAB-8531.

Bioinformatics processing

Bioinformatics processing followed detailed methods published elsewhere.[10] Raw sequences in FASTQ format were downloaded from the sequencing centre. Quality control of the raw sequences were performed using *FastQC* v0.118 to evaluate the overall quality. The raw sequences were manipulated using *TrimGalore* v0.6.2 to remove the adapter sequences and low-quality sequences. Next, clean reads were mapped to the human reference genome (GRCh38 *Homo sapiens* reference genome release 96) using *STAR* v2.7 aligner. To improve the quality of the data, only uniquely mapping reads with a mapping quality score 255 were counted. Post-alignment quality control was carried out on all aligned BAM files. *featureCounts* v1.6.5 was used to quantify the read counts of each gene for the subsequently differential expression analysis. The expression profile of each gene was expressed as read counts.

Differential expression analysis

The raw read counts were used as an input for *DESeq2* v1.26, an R/Bioconductor package for differential expression analysis. *DESeq2* normalizes the raw read counts based on the median of rations as described previously. Partial least squares discriminant analysis (PLS-DA), a supervised method implemented in *mixOmics* v6.10.2 was performed. PLS-DA was used to classify nine patients based on the *DESeq2* normalized counts for 18,426 genes. Two clusters in the PLS-DA plot consistently placed the nine samples into two groups. Recently, PLS-DA has been adapted to RNA-seq data to provide transcriptome expression landscape due to its ability to discriminate sample artefacts. Differentially expressed genes (DEGs) were conducted in a pairwise manner, using *DESeq2*. Genes achieving a false discovery rate (*FDR*) threshold below 0.05 were considered significantly differentially expressed. The full gene list is attached in Table S5. Volcano plot and heatmap of DEGs were constructed using the *ggplot2* package and '*pheatmap*' function implemented in *R*, respectively.

Table S1. Demographic and clinical characteristics of cohort.

	CHF (n=28)	CON (n=9)	*P*-value
Demographics			
Male sex [n (%)]	26 (93)	6 (67)	0.046
Age (years)	69.3±2.3	75.4±2.0	0.055
Weight (kg)	85.9±3.6	80.3±4.3	0.405
Height (m)	1.7±0.02	1.7±0.04	0.443
BMI (kg·m^2)	29.5±0.9	26.9±1.3	0.156
Clinical characteristics			
NYHA class [n (%)]			
I	2 (7)	9 (100)	<0.001
II	16 (57)	0 (0)	
III	10 (36)	0 (0)	
IHD [n (%)]	16 (57)	2 (22)	0.068
DCM [n (%)]	10 (36)	0 (0)	0.036
AF [n (%)]	10 (36)	3 (33)	0.896
CABG [n (%)]	5 (18)	1 (11)	0.633
HTN [n (%)]	11 (39)	4 (44)	0.784
COPD [n (%)]	0 (0)	1 (11)	0.074
LVEF (%)	28.6±2.4	51.4±1.5	<0.001
LVIDd (mm)	49.9±2.4	31.4±2.4	<0.001
Hb (g·L^{-1})	141.7±3.0	129.9±6.6	0.073
Na (mmol·L^{-1})	139.7±0.6	140.6±0.9	0.490
K (mmol·L^{-1})	4.6±0.1	4.5±0.3	0.629
Creatinine (µmol·mL^{-1})	98.1±4.9	84.3±2.8	0.123
eGFR (mL·min^{-1}·1.73 m^{-2})	64.8±3.8	71.1±5.1	0.395
Glucose (mmol·L^{-1})	9.0±3.2	5.2±5.1	0.495
HbA1c (mmol·mol^{-1})	32.9±5.1	26.8±6.7	0.540

Data are mean±SEM and were assessed by unpaired Student's *t*-test. BMI, body mass index; $\dot{V}O_{2peak}$, peak pulmonary consumption; NYHA, New York Heart Association; IHD, ischaemic heart disease; DCM, dilated cardiomyopathy; AF, atrial fibrillation; CABG, coronary artery bypass graft; HTN, hypertension; COPD, chronic obstructive pulmonary disease; LVEF, left ventricular ejection fraction; LVIDs, left ventricular internal diameter in systole; LVIDd, left ventricular internal diameter in diastole; Hb, haemoglobin; Na, serum sodium; K, serum potassium; eGFR, estimated glomerular filtration rate; NT-pro-BNP, N-terminal pro hormone B-type natriuretic peptide; HbA1c, glycated haemoglobin.

Table S2. Medications and device therapy.

	CHF (n=28)	CON (n=9)	*P*-value
Medications			
ACEi [n (%)]	22 (79)	2 (22)	0.002
Beta-blocker [n (%)]	27 (96)	1 (11)	<0.001
Furosemide equivalent dose (mg)	33.6 ± 6.4	8.9 ± 8.9	0.055
ARB [n (%)]	5 (18)	1 (11)	0.633
MRA [n (%)]	13 (46)	1 (11)	0.057
Statin [n (%)]	20 (71)	6 (67)	0.786
Antiplatelet [n (%)]	14 (50)	1 (11)	0.039
Metformin [n (%)]	6 (75)	0 (0)	0.129
Insulin [n (%)]	2 (7)	0 (0)	0.410
Sulphonylurea [n (%))]	3 (11)	0 (0)	0.306
Anticoagulant [n (%)]	7 (25)	5 (56)	0.218
Digoxin [n (%)]	4 (14)	0 (0)	0.230
Ivabradine [n (%)]	1 (4)	0 (0)	0.565
Device therapy			
PPM [n (%)]	0 (0)	9 (100)	<0.001
ICD [n (%)]	6 (21)	0 (0)	0.129
CRT [n (%)]	22 (79)	0 (0)	<0.001

Continuous variables are presented as mean±SEM, categorical variables as number (%) and were assessed by unpaired Student's t-test. ACEi, angiotensin-converting enzyme inhibitor; ARB, angiotensin receptor blocker; MRA, mineralocorticoid receptor antagonist; PPM, permanent pacemaker; ICD, implantable cardioverter defibrillator; CRT, cardiac resynchronisation therapy.

Table S3. Demographic and clinical characteristics for participants included in the RNAseq analysis.

	CHF (n=6)	CON (n=3)	*P*-value
Demographics			
Male sex [n (%)]	6 (100)	2 (67)	0.13
Age (years)	76.7 ± 3.3	80.2 ± 2.6	0.51
Weight (kg)	77.2 ± 7.5	74.5 ± 20.7	0.85
Height (m)	1.7 ± 0.05	1.7 ± 0.1	0.62
BMI ($kg\cdot m^2$)	27.6 ± 1.7	24.9 ± 1.9	0.37
Clinical characteristics			
NYHA class			
I	2 (33)	3 (100)	0.17
II	2 (33)	0 (0)	
III	2 (33)	0 (0)	
IHD [n (%)]	3 (50)	1 (33)	0.64
DCM [n (%)]	3 (50)	0 (0)	0.13
AF [n (%)]	3 (50)	1 (33)	0.64
CABG [n (%)]	2 (33)	1 (33)	1.0
HTN [n (%)]	3 (50)	3 (100)	0.13
COPD [n (%)]	0 (0)	1 (33)	0.13
LVEF (%)	26.3 ± 5.6	50.8 ± 3.0	0.006
LVIDd (mm)	56.5 ± 3.6	49.7 ± 2.4	0.26
Hb ($g\cdot L^{-1}$)	143.0 ± 3.7	114 ± 13.9	0.029
Na ($mmol\cdot L^{-1}$)	143.0 ± 1.0	138.7 ± 1.2	0.039
K ($mmol\cdot L^{-1}$)	4.3 ± 0.2	4.4 ± 0.7	0.88
Creatinine ($\mu mol\cdot mL^{-1}$)	96.7 ± 8.5	86.0 ± 7.0	0.45
eGFR ($mL\cdot min^{-1}\cdot 1.73\ m^{-}$	67.7 ± 6.9	69.0 ± 12.1	0.92
HbA1c ($mmol\cdot mol^{-1}$)	39.4 ± 1.2	44.0	-

Continuous variables are presented as mean ± SEM, categorical variables as number (%). BMI, body mass index; $\dot{V}O_{2peak}$, peak pulmonary consumption; NYHA, New York Heart Association; IHD, ischaemic heart disease; DCM, dilated cardiomyopathy; AF, atrial fibrillation; CABG, coronary artery bypass graft; HTN, hypertension; COPD, chronic obstructive pulmonary disease; LVEF, left ventricular ejection fraction; LVIDs, left ventricular internal diameter in systole; LVIDd, left ventricular internal diameter in diastole; Hb, haemoglobin; Na, serum sodium; K, serum potassium; eGFR, estimated glomerular filtration rate; NT-pro-BNP, N-terminal pro hormone B-type natriuretic peptide; HbA1c, glycated haemoglobin.

Table S4. Summary statistics of RNA sequencing, total number of reads generated, read mapping to the GRCh38 *Homo sapiens* reference genome, RNA integrity number (RIN), for nine RNA samples.

**Sample**	**Raw reads**	**Unique reads**	**Percentage of mapped reads**	**RIN score**
**SKM_C_1**	16216274	15022715	92.64	8
**SKM_C_2**	18364747	16835780	91.67	7.5
**SKM_C_3**	20400222	18670124	91.52	8.2
**SKM_HF_1**	14233566	13140795	92.32	8.6
**SKM_HF_2**	16437260	15135973	92.08	8.8
**SKM_HF_3**	14932640	13646610	91.39	7.6
**SKM_HF_4**	15333154	14116260	92.06	8.5
**SKM_HF_5**	14537174	13352387	91.85	8.9
**SKM_HF_6**	15308095	14092935	92.06	7.9

Table S5. Gene database. Please see Excel file.

Table S6. Demographic and clinical characteristics from two apparently distinct clusters of patients with CHF derived from the RNAseq analysis.

	Cluster 1 (1,3,4)	Cluster 2 (2,5,6)	*P*-value
Demographics			
Male sex [n (%)]	3 (100)	3 (100)	1.0
Age (years)	76.1 ± 12.6	77.2 ± 0.8	0.88
Weight (kg)	80.7 ± 7.2	73.7 ± 27.6	0.69
Height (m)	1.7 ± 0.1	1.6 ± 0.2	0.68
BMI (kg·m^2)	28.3 ± 2.9	26.8 ± 5.8	0.70
Clinical characteristics			
NYHA class [n (%)]			
I	0 (0)	2 (67)	0.14
II	1 (33)	1 (33)	
III	2 (67)	0 (0)	
IHD [n (%)]	2 (67)	1 (33)	0.41
DCM [n (%)]	1 (33)	2 (67)	0.41
AF [n (%)]	1 (33)	2 (67)	0.41
CABG [n (%)]	1 (33)	1 (33)	1.0
HTN [n (%)]	2 (67)	1 (33)	0.41
COPD [n (%)]	0 (0)	0 (0)	.
LVEF (%)	38.3 ± 5.8	14.2 ± 1.4	0.002
LVIDd (mm)	52.0 ± 7.8	61.0 ± 8.7	0.25
Hb (g·L^{-1})	138.7 ± 4.0	147.3 ± 11.5	0.29
Na (mmol·L^{-1})	144.3 ± 2.5	141.7 ± 2.1	0.23
K (mmol·L^{-1})	4.2 ± 0.5	4.3 ± 0.6	0.84
Creatinine (µmol·mL^{-1})	85.7 ± 22.1	107.7 ± 15.5	0.23
eGFR (mL·min^{-1}·1.73 m^{-2})	76.7 ± 19.1	58.7 ± 10.8	0.23
HbA1c (mmol·mol^{-1})	40.0 ± 3.6	38.5 ± 2.1	0.64

Data are mean±SEM and were assessed by unpaired Student's *t*-test. BMI, body mass index; $\dot{V}O_{2peak}$, peak pulmonary consumption; NYHA, New York Heart Association; IHD, ischaemic heart disease; DCM, dilated cardiomyopathy; AF, atrial fibrillation; CABG, coronary artery bypass graft; HTN, hypertension; COPD, chronic obstructive pulmonary disease; LVEF, left ventricular ejection fraction; LVIDs, left ventricular internal diameter in systole; LVIDd, left ventricular internal diameter in diastole; Hb, haemoglobin; Na, serum sodium; K, serum potassium; eGFR, estimated glomerular filtration rate; NT-pro-BNP, N-terminal pro hormone B-type natriuretic peptide; HbA1c, glycated haemoglobin.
